# De Novo Design of Polymeric Carrier to Photothermally Release Singlet Oxygen for Hypoxic Tumor Treatment

**DOI:** 10.34133/2019/9269081

**Published:** 2019-05-15

**Authors:** Tianci Huang, Menglong Zhao, Qi Yu, Zheng Feng, Mingjuan Xie, Shujuan Liu, Kenneth Yin Zhang, Qiang Zhao, Wei Huang

**Affiliations:** ^1^Key Laboratory for Organic Electronics and Information Displays & Jiangsu Key Laboratory for Biosensors, Institute of Advanced Materials (IAM), Jiangsu National Synergetic Innovation Center for Advanced Materials (SICAM), Nanjing University of Posts and Telecommunications (NUPT), Nanjing 210023, China; ^2^Shaanxi Institute of Flexible Electronics (SIFE), Northwestern Polytechnical University (NPU), Xi'an 710072, Shaanxi, China

## Abstract

Intratumoral hypoxia extremely limits the clinic applications of photodynamic therapy (PDT). Endoperoxides allow thermally releasing singlet oxygen (^1^O_2_) in a defined quantity and offer promising opportunities for oxygen-independent PDT treatment of hypoxic tumors. However, previous composite systems by combining endoperoxides with photothermal reagents may result in unpredicted side effects and potential harmful impacts during therapy* in vivo*. Herein, we de novo design an all-in-one polymer carrier, which can photothermally release ^1^O_2_. The strategy has been demonstrated to effectively enhance the production of ^1^O_2_ and realize the photodamage in vitro, especially in hypoxic environment. Additionally, the polymer carrier accumulates into tumor after intravenous injection via the enhanced permeation and retention effects and accelerates the oxygen-independent generation of ^1^O_2_ in tumors. The oxidative damage results in good inhibitory effect on tumor growth. Realization of the strategy* in vivo* paves a new way to construct photothermal-triggered oxygen-independent therapeutic platform for clinical applications.

## 1. Introduction

Photodynamic therapy (PDT) has become an emerging noninvasive and selective cancer therapeutic modality, in which light triggers energy transfer between triplet excited states of photosensitizers and molecular oxygen to generate cytotoxic singlet oxygen (^1^O_2_) leading to apoptotic cell death [1–3]. It has been used for age-related macular degeneration, viral infection, atherosclerosis, and malignant cancers [4, 5]. However, intratumoral hypoxia severely limits its clinic applications owing to insufficient generation of ^1^O_2_. Furthermore, the consumption of oxygen during PDT treatment aggravates the hypoxic environment, further limiting the therapeutic outcome [6, 7]. To address this problem, oxygen-sufficient materials or oxygen-independent photosensitizers to generate reactive oxygen species are developed [8–19].

Polycyclic aromatic hydrocarbons are the most reliable organic compounds to cleanly supply ^1^O_2_ in a defined quantity without side reaction [20–24]. They can trap ^1^O_2_ yielding endoperoxides (EPOs) and release ^1^O_2_ upon elevating the temperature, which provides a new and powerful concept in ^1^O_2_ delivery for the treatment of hypoxic tumors. Till now, only few reports have developed the composite systems by combining EPOs with photothermal reagents (such as gold nanorods) to release ^1^O_2_ in cancer cells [25, 26]. Although they realized photothermal-triggered oxidative damage of cancer cells in* in vitro* experiments, the composite structures may result in unpredicted side effects and potential harmful impacts on biological environments during therapy* in vivo*. Moreover, the utilization of gold nanorods substantially increases the difficulty of clearance from the body and leads to long-term toxicity towards healthy tissues and organs [27]. In this regard, it is highly desirable to de novo design an all-in-one strategy without the above-mentioned concerns.

**Figure 1:**
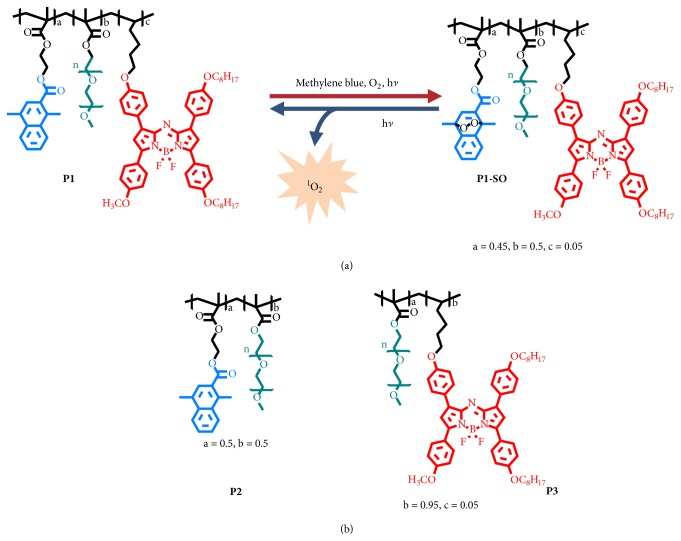
*Scheme illustration of the polymeric carrier serving as a PDT platform in vivo*. (a) Mechanism of capture and release of ^1^O_2_ by the polymeric carrier, a = 0.45, b = 0.5, and c = 0.05. (b) The structures of** P2** (a = 0.5, b = 0.5) and** P3 **(b = 0.95, c = 0.05).

Herein, we propose a novel all-in-one polymer carrier (**P1**), composed of 1,4-dimethylnaphthalene (**DMN**), aza-BODIPY (**B1**), and hydrophilic polyethylene glycol (PEG) (Figure 1(a)), in which,** DMN** is able to deliver ^1^O_2_ into hypoxic tumors via reversible transformation between the naphthalene and endoperoxide forms, while** B1** serves as not only an excellent organic photothermal agent, but also potential imaging reagents* in vivo* because of good physiological stability and near-infrared absorption and luminescence [28]. Taking advantages of oxygen-independence during the ^1^O_2_ generation process, the ^1^O_2_ loaded polymer** P1-SO** can be a good candidate to overcome the resistance of PDT caused by tumor hypoxia. In* in vivo* experiments,** P1-SO** was injected into tumor-bearing mice through tail vein; it accumulated in the tumor tissues, owing to the enhanced permeation and retention (EPR) effect. Moreover, laser irradiation on the** P1-SO**-injected mouse significantly restrained the growth of tumors, resulted from the oxidative damage towards hypoxic tumor. These results highlighted the excellent therapeutic effect of the designed all-in-one polymeric ^1^O_2_ carrier.

## 2. Results

### 2.1. Design, Synthesis, and Characterization of Polymers

1,4-Dimethylnaphthalene was selected as the ^1^O_2_ carrier, owing to the high capability to trap ^1^O_2_. Structural modification at the C2-position of the naphthalene rings allows easily controlling of ^1^O_2_ release. Aza-BODIPY (**B1**), which has strong NIR absorption and emission, was chosen as both the photothermal agent and imaging dye. Hydrophilic polyethylene glycol was introduced in the polymer to improve the water solubility and EPR effect. The monomer** DMN-acryl **has been synthesized according to the previous work [26]. The monomer** B1** was synthesized in four steps (See in the Supplementary Material). Firstly, compounds** 3** and** 5** were obtained through an aldol/dehydration reaction and then Michael addition reaction. Compound** 6** was directly transformed by** 3** and** 5**.** B1** was obtained by BF_2_ chelation step of** 6**.** P1**-**P3** were synthesized via radical polymerization. To balance the capacity for loading ^1^O_2_ and guarantee the thermal effects of the polymer, the molar ratios of** DMN** and** B1** in a polymer molecule were fixed to 45% and 5%, respectively. Endoperoxides of** DMN** and ^1^O_2_ loaded polymer (**P1-SO**) were formed in the presence of a commercial photosensitizer, methylene blue (**MB**), under the irradiation, and then** MB** could be removed easily by dialysis. The polymer dots were obtained by self-assembly due to their amphiphilic structures with hydrophobic** DMN** and** B1** units and hydrophilic PEG as side chain.

The model polymers** P2** and** P3** were also obtained (Figure 1(b)). The detailed synthesis procedures of the polymer dots with** DMN** and aza-BODIPY pendants were illustrated in supporting information. The monomers and polymers were characterized by NMR spectra, gel permeation chromatography (GPC), transmission electron microscope (TEM) images, dynamic light scattering (DLS), and matrix-assisted laser desorption ionization time of light mass spectrometry (MALDI-TOF MS).

**Figure 2:**
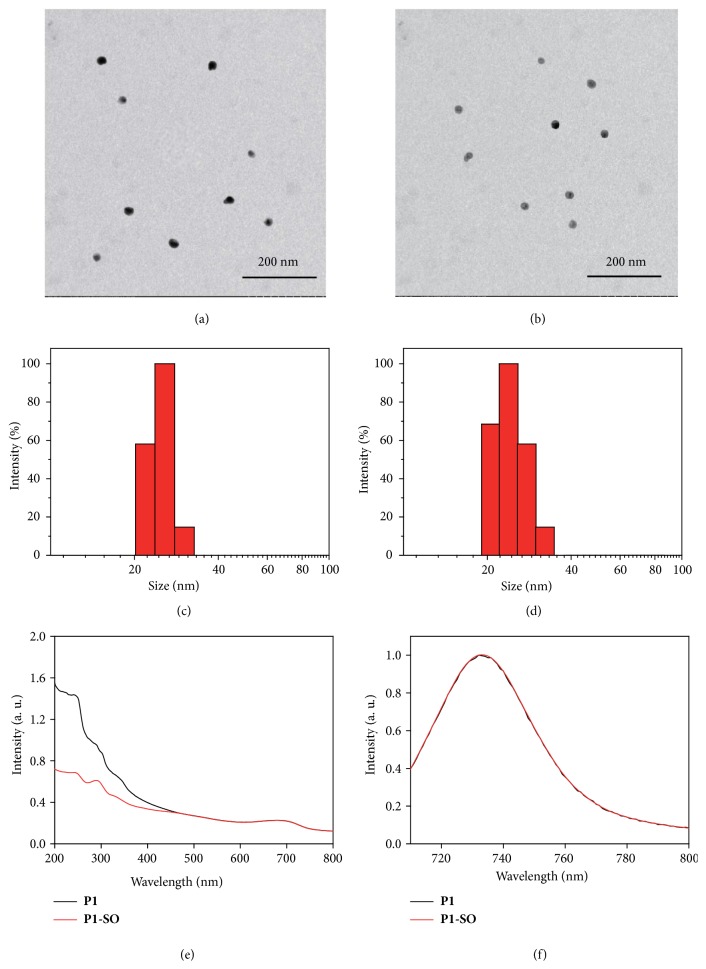
*Morphology, diameter, and photophysical properties of* ***P1*** *and* ***P1-SO***. TEM images of** P1** (a) and** P1-SO** (b). Size distribution of** P1** (c) and** P1-SO** (d) by DLS. Absorption spectra (e) and emission spectra (f) of** P1** and** P1-SO** in DMSO.

The number-average molecular weights (*M*n) of** P1**-**P3** were 19674, 21024, and 16024, respectively. The polydispersity index (*M*w/*M*n) of** P1**-**P3** was 1.20, 1.31, and 1.24, respectively. A uniform spherical morphology of** P1** dots was clearly revealed by TEM images (Figure 2(a)). The diameters of** P1** dots were estimated to be around 20 nm. DLS indicated that the** P1** dots were well-dispersed in water and had an average hydrodynamic size of 23 nm (Figure 2(c)), which contributed to be enriched in tumor tissues via EPR effect [29]. After loading ^1^O_2_, the results of TEM and DLS of** P1-SO** dots remain almost the same as those of** P1** dots (Figures 2(b) and 2(d)), demonstrating that capture of ^1^O_2_ did not change the morphology, particle size, or dispersity of the polymer dots.

### 2.2. Photophysical, Photothermal, and Photodynamic Properties of** P1-SO** Dots

The photophysical properties of** P1** and** P1-SO** dots were investigated by UV-Vis absorption and emission spectra. As illustrated in Figure 2(e), the absorption spectra of** P1** dots displayed a strong absorption at 243 nm and a weak absorption at 689 nm, which were consistent with the absorption of** DMN** and** B1** monomer, respectively. After capture of ^1^O_2_, the destruction of conjugated structure of** DMN** leaded to a sharp decrease at 243 nm in the UV-Vis absorption spectra [26]. In addition,** P1** dots and** P1-SO** dots showed almost the same emission maxima at 730 nm (Figure 2(f)), which was attributed to the luminescence of** B1** (see [Supplementary-material supplementary-material-1] in the Supplementary Material) and negligibly affected by the ^1^O_2_ loading.

**Figure 3:**
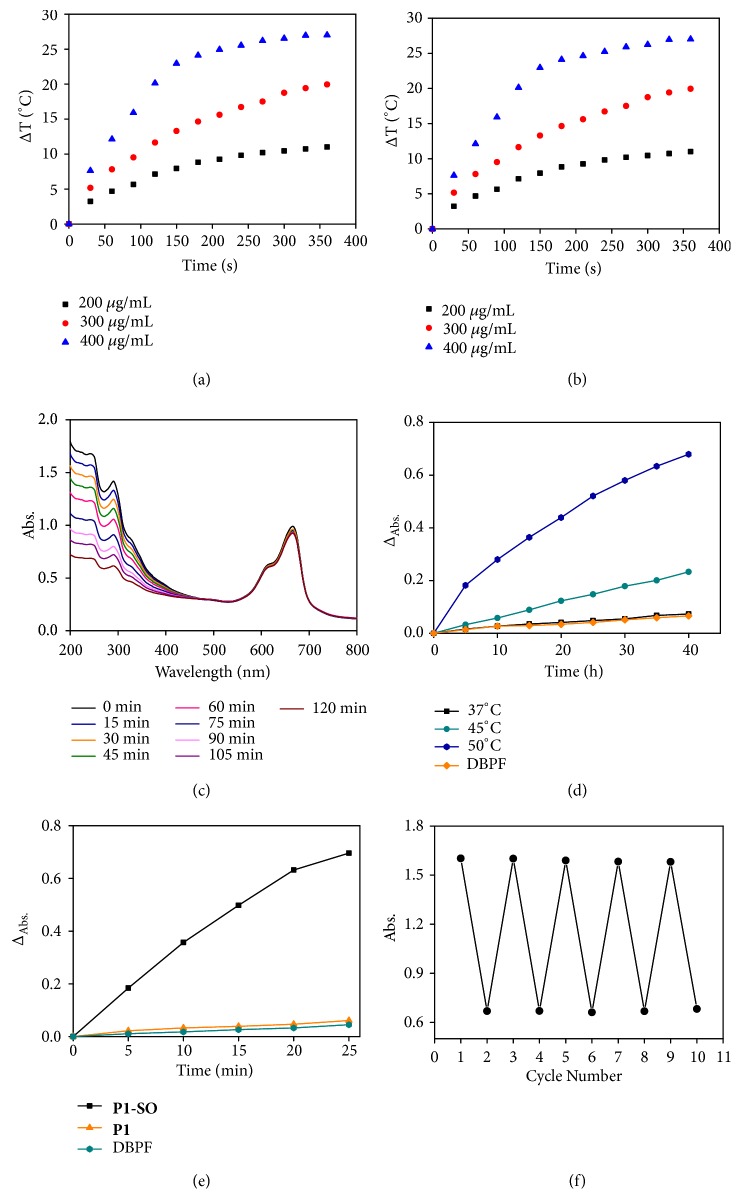
*Photothermal effects, capture, and release of* ^*1*^ *O*_*2*_ *of* ***P1*** *and* ***P1-SO***. (a) and (b) Temperature elevation of** P1** and** P1-SO** at different concentrations under irradiation (690 nm, 400 mW/cm^2^). (c) Absorption spectra of the mixture of** P1** (300 *μ*g/mL) and** MB** (10 *μ*M) under different irradiation time (660 nm, 4 mW/cm^2^). (d) Δ_Abs_ of DPBF under different temperatures in mixture solution of** P1-SO** and DBPF. (e) Δ_Abs_ of DPBF in the mixture solution of** P1-SO** or** P1** and DBPF under irradiation (690 nm, 400 mW/cm^2^). (f) The cycle number of the capture and release of ^1^O_2_. Δ_Abs_ = A_t_ − A_0_, A_t_ was the absorption at different irradiation time and A_0_ was the absorption without irradiation.

Thermal effects were the key factor to trigger the releasing of ^1^O_2_. Therefore, the photothermal performance of monomer** B1**,** P1**, and** P1-SO** was carried out via thermal infrared imager (FLIR E40). The temperature change was recorded at different concentration in dimethylsulfoxide (DMSO) under continuous exposure to irradiation (690 nm, 400 mW/cm^2^). As shown in [Supplementary-material supplementary-material-1] in the Supplementary Material and Figures 3(a) and 3(b), the increasing concentration resulted in the elevation of temperature. After irradiating for 360 s, the temperatures increased by 21.5°C, 20.0°C and 19.8°C for** B1** (40 *μ*M),** P1** (300 *μ*g/mL), and** P1-SO** (300 *μ*g/mL), respectively, demonstrating that the polymer containing** B1** units displayed good photothermal effects.

To investigate the ^1^O_2_ capture ability of** P1**,** MB** was added to the DMSO solution of** P1**. As shown in Figure 3(c), when the mixture solution was irradiated by a 660 nm laser (4 mW/cm^2^), the absorption band at 243 nm decreased gradually with the extension of irradiation time, but the absorption of** MB** at 665 nm remained unchanged, indicating that ^1^O_2_ sensitized by** MB** was captured by** P1** to form** P1-SO**. After removing** MB**, the abilities of ^1^O_2_ release of** P1-SO** were studied at different temperatures (Figure 3(d)). When the temperature was kept at 37°C, rare ^1^O_2_ was released according to the negligible response of the absorption band of the ^1^O_2_ indicator, 1,3-diphenylisobenzofuran (DPBF). In contrast, a sharp decrease of absorption band was observed when the temperature was increased to 50°C, indicating of a large amount of ^1^O_2_ generated. These results demonstrated that elevated temperature facilitated the release of ^1^O_2_. Moreover, to investigate the release of ^1^O_2_ under photothermal stimulation, the generation of ^1^O_2_ of** P1-SO** was measured under irradiation of laser (690 nm, 400 mW/cm^2^) in air and hypoxia environment that was produced by bubbling with nitrogen gas (Figure 3(e)). In the DMSO solution of** P1-SO** and DPBF, obvious decrease of the absorption band ascribed to DPBF was observed in both air and hypoxia environments, indicating that the oxygen levels had negligible influence for the ^1^O_2_ release. On the other hand, in the control group, when** P1** was used instead of** P1-SO**, it was hardly able to generate ^1^O_2_ even in air since ^1^O_2_ was not trapped in the** DMN** units and heavy atom-free** B1** had no ability to generate ^1^O_2_. Furthermore, to demonstrate the reversibility of the capture and release of ^1^O_2_ for** P1-SO**, we added** MB** to the DMSO solution containing** P1** and exposed the mixture under light irradiation (660 nm, 4 mW/cm^2^). The absorption band at 243 nm was decreased after the light irradiation (660 nm, 4 mW/cm^2^) for 120 min owing to the capture of ^1^O_2._ When the light irradiation (690 nm, 400 mW/cm^2^) was prolonged for 25 min, the absorption band at 243 nm was increased because of the^1^O_2_ releasing.** MB** was not removed in this process. The reversible performance was displayed for 5 cycles (Figure 3(f)). The model polymer** P2** also had ability to trap ^1^O_2_ to form** P2-SO**, but** P2-SO** was unable to release ^1^O_2_ under irradiation owing to the lack of photothermal agents (see Figures [Supplementary-material supplementary-material-1]-[Supplementary-material supplementary-material-1] in the Supplementary Material), while** P3** could neither trap ^1^O_2_ nor generate ^1^O_2_ (see [Supplementary-material supplementary-material-1] in the Supplementary Material).

All the results indicated that the photothermal effects of** B1** could provide enough heat to trigger ^1^O_2_ release and** P1-SO** had excellent phototoxicity effects in solution. More importantly, oxygen is unnecessary in ^1^O_2_ release process of** P1-SO** compared to conventional photosensitizers, revealing enormous potential for improving the therapeutic effects of hypoxia-associative PDT.

### 2.3. Anticancer Investigation In Vitro

**Figure 4:**
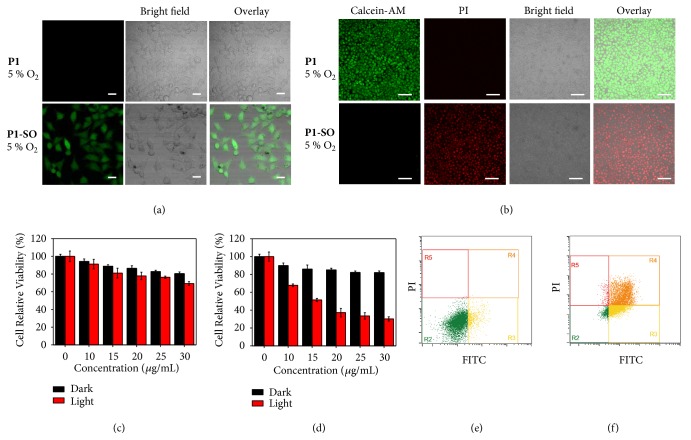
*In vitro evaluation with* ***P1*** *and* ***P1-SO***. (a) ROS generation in HeLa cells with DCFH-DA, cells were incubated with** P1** or** P1-SO** for 2 h under 5% oxygen level under irradiation for 6 min (690 nm, 400 mW/cm^2^) (scale bar, 50 nm). (b) Calcein-AM and PI stained HeLa cells were incubated with** P1** and** P1-SO** and then exposed under 690 nm laser irradiation (400 mW/cm^2^) for 6 min under 5% oxygen level (scale bar, 100 *μ*m). (c) and (d) MTT assay of** P1** and** P1-SO** under 5% oxygen level with and without irradiation. (e) and (f) Flow cytometry quantification of apoptosis of HeLa cells incubated with** P1** and** P1-SO** under 5% oxygen level with 690 nm laser irradiation (400 mW/cm^2^).

To demonstrate the feasibility of the polymer carrier to generate ^1^O_2_* in vitro*, 2,7-dichlorifluoresceindiacetate (DCFH-DA), which can be oxidized to 2,7-dichlorofluorescein (DCF) by intracellular ROS in live cells, was utilized as a ROS tracer agent. The laser-scanning confocal luminescence microscopy was employed to investigate the ROS generation in HeLa cells. Upon irradiation at 690 nm, weak luminescence in the cells was observed under 21% (see [Supplementary-material supplementary-material-1] in the Supplementary Material) and 5% oxygen concentration (Figure 4(a)) when the cells were incubated with** P1**. But bright green luminescence of DCF was exhibited in the cells incubated with** P1-SO** under 21% and 5% oxygen concentration. Without light irradiation, the generation of ROS hardly occurred in the cells incubated with** P1-SO**, since the physiological temperature (37°C) was relatively low so that it could not trigger the rapid release of ^1^O_2_ from endoperoxides (see [Supplementary-material supplementary-material-1] in the Supplementary Material). The control group containing the cells only incubated with DCFH-DA displayed negligible luminescence in the absence and presence of irradiation at 690 nm (see [Supplementary-material supplementary-material-1] in the Supplementary Material). These results demonstrated that the intracellular release of ^1^O_2_ from endoperoxides could be accelerated by photothermal effects of** B1** in** P1-SO**.

The methyl thiazolyl tetrazolium (MTT) assay was used to evaluate the cytotoxicity of the polymer carrier towards HeLa cells. HeLa cells were incubated with different concentrations of** P1** or** P1-SO** at 37°C for 24 h in dark. Low dark cytotoxicity of** P1** and** P1-SO** were shown under 5% and 21% oxygen. When the** P1** incubated cells were treated by 690 nm laser, the cell viability was relatively high for cells in 21% or 5% oxygen concentrations, indicating low toxicity of** P1** (Figures 4(c) and [Supplementary-material supplementary-material-1] in the Supplementary Material). Additionally, the cells incubated with ^1^O_2_ loading** P1-SO** showed relatively lower cell viability in 21% (see [Supplementary-material supplementary-material-1] in the Supplementary Material) or 5% (Figure 4(d)) oxygen compared to the group of** P1**-treated cells under irradiation, revealing that the ^1^O_2_ release induced the oxidative damage and was the dominant reason to kill cells.

To study the therapy performance via cell apoptosis assay, Calcein-AM and PI were used to label the living and dead cells as indicators by staining the cytoplasm with green fluorescent AM and the nucleus with red fluorescent PI, respectively. The cells remained alive in dark. Under irradiation, the cells incubated with** P1** remained alive even under 21% oxygen, whereas the cells incubated with** P1-SO** were dead under either 21% or 5% oxygen concentration (Figures 4(b) and [Supplementary-material supplementary-material-1] in the Supplementary Material). Without irradiation, the cells treated with** P1** or** P1-SO** mostly remained alive under 21% and 5% oxygen levels (see [Supplementary-material supplementary-material-1] in the Supplementary Material). These results confirmed that the oxidative damage could be achieved by** P1-SO** in hypoxic cancer cells. To further determine the cell population at different stages of apoptosis, the flow cytometry experiments were performed (Figures 4(e) and 4(f) and [Supplementary-material supplementary-material-1] in the Supplementary Material). Under irradiation by laser at 690 nm, the cells incubated with** P1** were still alive but those incubated with** P1-SO** were dead after 6 hours under 5% oxygen condition (Figures 4(e) and 4(f)). All the results indicated that photothermal effect of** P1-SO** triggered the ROS generation and the oxidative damage dominated the therapeutic effects, encouraging us to investigate the potential application of** P1-SO** for cancer therapy in hypoxia environments.

### 2.4. Anticancer Investigation In Vivo

**Figure 5:**
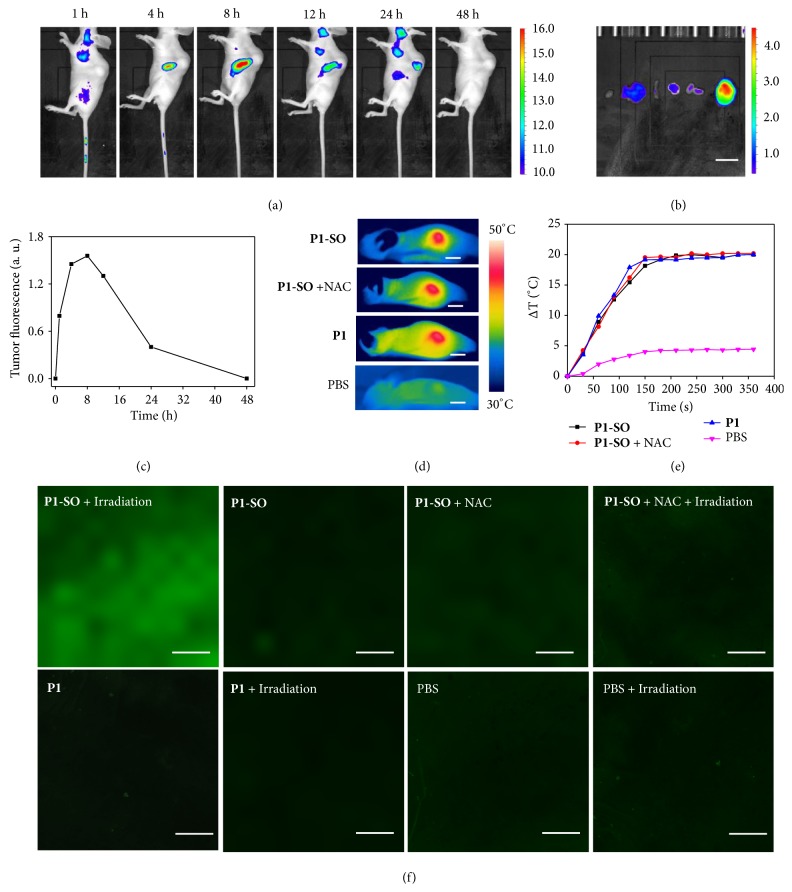
*In vivo photothermal effects and generation of* ^*1*^ *O*_*2*_ *of* ***P1*** *and* ***P1-SO***. (a)* In vivo* fluorescence imaging of the HeLa tumor-bearing mouse at different time points after tail intravenous injection of** P1-SO**. (b) Fluorescence imaging of main organs after tail intravenous injection of** P1-SO** 8 h (scale bar, 1 cm). (c) The fluorescence intensity of tumor at different time. (d) Photothermal imaging of the mice bearing HeLa tumor treated with different treatments under irradiation (690 nm, 400 mW/cm^2^) (scale bar, 1 cm). (e) Temperature changes of mice tumors with different treatments under irradiation for 6 min (690 nm, 400 mW/cm^2^). (f) DCFH-DA staining at the tumors of mice with different treatments under irradiation for 6 min (690 nm, 400 mW/cm^2^) (scale bar, 250 *μ*m).

To test the* in vivo* behaviors of** P1-SO**, the polymer carrier was intravenously injected into the HeLa tumor-bearing mice, and then their biodistributions were evaluated at 1, 4, 8, 12, 24, and 48 h after injection (Figure 5(a)). After injection of** P1-SO** for 1 h, the gradually enhanced fluorescence at the tumors was observed. A maximized distribution was exhibited in tumor at 8 h after injection compared with other major organs (Figure 5(b)), demonstrating that the highest level of** P1-SO** was accumulated in tumor. After 48 h, no fluorescence was observed in tumor, revealing the metabolism of** P1-SO** with time prolonging (Figure 5(c)). Hence, the PDT treatments will be carried out at 8 h after injection of** P1-SO**. After the anticancer treatments,** P1-SO** can be eliminated from the body. These results indicated good capacity of the polymer dots to accumulate in tumor, owing to the EPR effect mediated by appropriate particle size.

To prove the photothermal effects of the polymer carrier* in vivo*, four groups of tumors bearing mice were investigated after 8 h after injection (Figure 5(d)). Upon irradiation at 690 nm (400 mW/cm^2^) by a FLIR camera, mice injected with** P1-SO** and** P1** showed temperature increase to about 48°C in the tumor area. Similar temperature increase was also observed when the mouse was injected with** P1-SO** and* N*-acetyl-*L*-cysteine (NAC), which is a ROS-scavenger. In the control experiment where the mouse was injected with PBS, the temperature was almost kept at 37°C (Figure 5(e)). To investigate the ability of ROS generation in tumor, the tumor biopsies of mice with different treatments were studied by confocal imaging (Figure 5(f)). The* in vivo *generation of ^1^O_2_ from** P1-SO** under irradiation was observed using DCFH-DA as the indicator. After intravenous injection of** P1-SO** for 8 h, NAC was injected into the tumor. With a 690 nm laser irradiation (400 mW/cm^2^) for 6 min, no luminescence of DCF was found, and** P1** treated tumor also displayed no ^1^O_2_ generation under irradiation. These results prove that** P1-SO** has strong ability to generate ^1^O_2_* in vivo* under irradiation.

**Figure 6:**
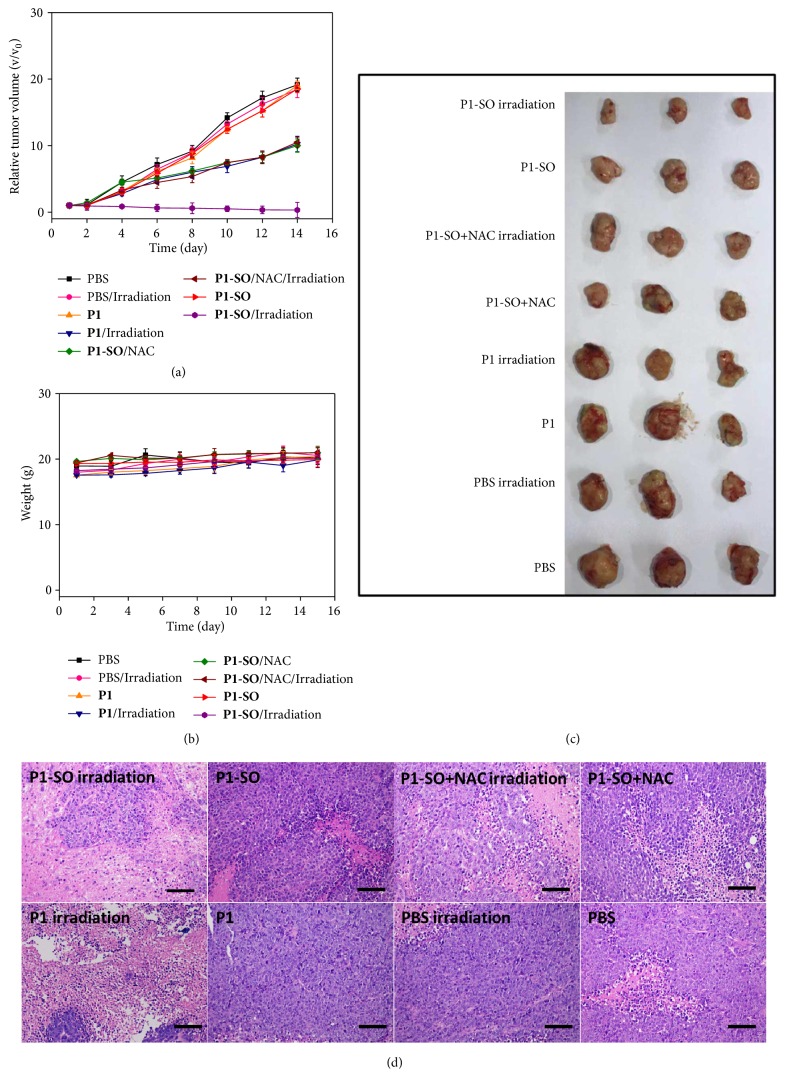
*In vivo treatments of* ***P1*** *and* ***P1-SO***. (a) Relative tumor volume changes of mice with different treatments. Relative tumor volume was calculated by the (b) Body weight changes of mice with different treatments. (c) Photograph of the tumors extracted from the mice. (d) H&E-stained tumor sections harvested from mice after different treatments (scale bar, 100 *μ*m).

To investigate the* in vivo* PDT efficacy,** P1-SO** or** P1** was intravenously injected into the mice bearing HeLa tumors of 100-300 mm^3^ in the presence or absence of NAC, followed by irradiation at 690 nm laser for 6 min (400 mW/cm^2^). The tumor size and weight of the mice were recorded every 2 days (Figures 6(a)–6(c)). As a control, the mice injected with PBS showed a 19-fold increase of tumor volumes regardless of irradiation, indicating that the irradiation of laser hardly had evident influence to the growth of tumors. Without irradiation,** P1** and** P1-SO** treated mice also displayed similar tumor growth rate compared to those injected with PBS, demonstrating negligible anticancer efficacy of** P1** and** P1-SO** due to their low dark cytotoxicity. When tumors were exposed to irradiation,** P1** treated tumors showed a 10-fold increase of tumor volumes, but clear shrink of tumors was found on the mice injected with** P1-SO** after 2 weeks, indicating that the photothermal effect of** B1** was unable to eliminate tumor but can trigger the release of ^1^O_2_ in** P1-SO**. Importantly, ^1^O_2_ generation by** P1-SO** caused irreversible oxidative damage towards tumor and inhibited the growth of tumor. To further prove the influence of ^1^O_2_ generation for tumor therapy, the ROS­scavenger NAC was intratumorally injected into the** P1-SO** treated mice before therapy. The tumors did not stop growing and displayed 11-fold increases of tumor volumes after 14 days under irradiation. All the photographs of the mice after treatments were shown in [Supplementary-material supplementary-material-1] in the Supplementary Material. These results demonstrated that the released ^1^O_2_ played a dominant role in the therapeutic process.

To further evaluate the detail anticancer efficacy of** P1** and** P1-SO**, the proliferation and morphology of the tumors and organs were investigated by hematoxylin & eosin (H&E) staining (Figure 6(d)). Firstly, obvious tumor damage was not observed in group PBS with or without irradiation. Few tumor cells were found in the group of** P1-SO** with irradiation, indicating an ideal ability to release ^1^O_2_ of** P1-SO** under irradiation. A small amount of tumor necrosis displayed in group** P1-SO** + NAC and** P1** with irradiation owing to the photothermal effects. No distinct tumor damage was found in** P1-SO** or** P1** without irradiation, which indicated that irradiation played a key role in the therapeutic process. These results demonstrated that tumor damage caused by** P1-SO** under irradiation was mainly attributed to oxidative damage with few photothermal effects. In addition,** P1-SO** exhibited negligible influence to the normal tissues (such as heart, liver, spleen, lung, and kidney) during the therapeutic process (see [Supplementary-material supplementary-material-1] in the Supplementary Material).

## 3. Discussion

In this study, a novel all-in-one polymeric ^1^O_2_ carrier, which can rapidly release cytotoxic ^1^O_2_ in cancer cells under photothermal stimulation, was developed to overcome the restriction of hypoxic tumor during PDT process* in vivo*. The capture of ^1^O_2_ was attributed to the** DMN** units and the release of ^1^O_2_ was triggered by the photothermal effect of** B1** under NIR light irradiation. The strategy has been demonstrated to effectively enhance the production of ^1^O_2_* in vitro* and realize the photodamage to cancer cells, especially in hypoxic environments. Additionally, introduction of near-infrared excitable** B1** facilitates the potential imaging-guided therapy* in vivo*. The polymer dots accumulate into tumor after intravenous injection via EPR effect and accelerate the oxygen-independent generation of ^1^O_2_. The oxidative damage towards tumor results in good inhibitory effect on tumor growth* in vivo*. The realization of this concept* in vivo* not only is a huge boost to the novel thermal-triggered PDT strategy, but also provides a valuable means to construct photothermal-triggered oxygen-independent therapeutic platform for clinical applications.

## 4. Materials and Methods

### 4.1. Materials

All reagents and starting materials were purchased from commercial sources and used without further purification. All aqueous solutions were prepared by using deionized water.

### 4.2. Instruments

NMR spectra (^1^H: 400 MHz, ^13^C: 100 MHz) were recorded on a Bruker ACF400 spectrometer. Tetramethylsilane (TMS) was used to report chemical shifts. The number-average molecular weight (*M*_n_) of the polymers was characterized in tetrahydrofuran (THF) by gel permeation chromatography (GPC) using polystyrene as standard. UV-visible absorption spectra were obtained via a Shimadzu UV-3600 UV/Vis/NIR spectrophotometer. Emission spectra were obtained with Edinburgh FL 920 spectrophotometer. The particle size and morphology of polymer dots were characterized by the transmission electron microscope (TEM, JEOL JEM-2100, 200 kV). The average hydrodynamic size and zeta potential of polymer dots were measured via dynamic light scattering (DLS) on a zeta particle size analyzer (Brookhaven 90Plus). Oxygen concentration was controlled by flow counters (HORIBA STEC, SEC-E40JS, 60 SCCM). The excitation light source used to generate ^1^O_2_ and photothermal effect were MW-GX-660/2000mW and MW-GX-690/2000mW laser. Temperature was measured by a thermal infrared imager (FLIR E40). The power density meter is VLP-2000 laser power meter.* In vivo* and* vitro* imaging were measured by small animals living fluorescence imaging system IVIS LUMINA K/IVIS LUMINA K. Cell viability was measured with an enzyme-linked immune sorbent assay (ELISA) reader. Confocal luminescence images were carried out by a laser-scanning confocal microscopy (Olympus Fluo view FV1000) equipped with 20× objective lens. Photographs of the mice were taken with a Cannon EOC 400D digital camera.

### 4.3. Animals and Tumor Model

The athymic female nude mice were purchased from Comparative Medicine Centre of Yangzhou University (Permit number: SCXK(SU)2017-0007). HeLa cells (about 10^6^ per mouse) were injected into nude mice. The mice bearing HeLa tumors were treated when the tumor volumes were about 100 mm^3^.

### 4.4. In Vivo Therapy

24 mice were divided into 8 groups averagely.** P1-SO**,** P1** (300 *μ*g/L, 100 *μ*L), or PBS were injected. After 8 h, the mice were exposed to a 690 nm laser (400 mW/cm^2^) for 6 min or not. The weight and tumor volumes were recorded every two days. Volume of tumors was calculated by equation: volume = length × width^2^/2. The relative tumor volume = v/v_0_, v was the tumor volume at different day, v_0_ was the tumor volume at first day. All the mice were sacrificed after treatments and tumors and main organs (heart, liver, spleen, lung, and kidney) were fixed by using 4% formalin solution for further histomorphological analysis.

## Data Availability

All data needed to evaluate the conclusions in the paper are present in the paper and/or the Supplementary Materials. Additional data related to this paper may be requested from the authors.

## Supplementary Materials

Supplementary Materials Figure S1: the UV-Vis absorption and emission spectra of** B1**. Figure S2: the photothermal properties of** B1**. Figure S3: the singlet oxygen trapping properties of** P2**. Figure S4: the singlet oxygen generation of** P2 **and** P2-SO**. Figure S5: the singlet oxygen generation of** P3**. Figure S6: the ROS generation of** P1** and** P1-SO** under irradiation* in vitro*. Figure S7: the ROS generation of** P1** and** P1-SO** without irradiation* in vitro*. Figure S8: the ROS generation without photosensitizers* in vitro*. Figure S9: the cytotoxicity of** P1**. Figure S10: the cytotoxicity of** P1-SO**. Figure S11: the PDT effects of** P1** and** P1-SO** under irradiation* in vitro*. Figure S12: the PDT effects of** P1** and** P1-SO** without irradiation* in vitro*. Figure S13: flow cytometry of PDT effects under hypoxia. Figure S14: photographs of the mice. Figure S15: H&E stained of the main organs.
