# Supplementary material for: De Novo Design of Polymeric Carrier to Photothermally Release Singlet Oxygen for Hypoxic Tumor Treatment
Source: Research (Wash D C). 2019 May 15;2019:9269081. doi: 10.34133/2019/9269081 (PMC6750110; doi:10.34133/2019/9269081)
Supplement: Supplementary Materials — Figure S1: the UV-Vis absorption and emission spectra of B1. Figure S2: the photothermal properties of B1. Figure S3: the singlet oxygen trapping properties of P2. Figure S4: the singlet oxygen generation of P2 and P2-SO. Figure S5: the singlet oxygen generation of P3. Figure S6: the ROS generation of P1 and P1-SO under irradiation in vitro. Figure S7: the ROS generation of P1 and P1-SO without irradiation in vitro. Figure S8: the ROS generation without photosensitizers in vitro. Figure S9: the cytotoxicity of P1. Figure S10: the cytotoxicity of P1-SO. Figure S11: the PDT effects of P1 and P1-SO under irradiation in vitro. Figure S12: the PDT effects of P1 and P1-SO without irradiation in vitro. Figure S13: flow cytometry of PDT effects under hypoxia. Figure S14: photographs of the mice. Figure S15: H&E stained of the main organs. [file 9269081.f1.docx]

Supporting Information

De Nove Design of Polymeric Carrier to Photothermally Release Singlet Oxygen for Hypoxic Tumor Treatment

**Tianci Huang^1†^, Menglong Zhao^1†^, Qi Yu^1†^, Zheng Feng^1^, Mingjuan Xie^1^, Shujuan Liu^1^, Kenneth Yin Zhang^1^, Qiang Zhao^1*^, and Wei Huang^1,2*^**

^1^Key Laboratory for Organic Electronics and Information Displays & Jiangsu Key Laboratory for Biosensors, Institute of Advanced Materials (IAM), Jiangsu National Synergetic Innovation Center for Advanced Materials (SICAM), Nanjing University of Posts and Telecommunications (NUPT), Nanjing 210023, P. R. China.

^2^Shaanxi Institute of Flexible Electronics (SIFE), Northwestern Polytechnical University (NPU), Xi'an 710072, Shaanxi, P. R. China.

**^*^**Correspondence should be addressed to Qiang Zhao; iamqzhao@njupt.edu.cn and Wei Huang; provost@nwpu.edu.cn.

^†^These authors contributed equally to this work

1. **Synthesis and characterization of P1-P3**

**Synthesis of DMN-acryl:**

**DMN-acryl** was prepared by following literature procedure.^1^

**Synthesis of compound 1:**

4-Hydroxybenzaldehyde (3.403 g, 25 mmol) and 4-methoxyacetophenone (3.754 g 25 mmol) were dissolved in 100 mL alcohol. 10 mL NaOH (aq) (10%) was added into the mixture and then stirred at room temperature for 12 h. Then the mixture was filtered and washed with cold ethanol. The obtained yellow solid was used directly in the next step.

**Synthesis of compound 2:**

Compound **1** (5.086 g 20 mmol) was added to a mixture of 6-bromohex-1-ene (16.306 g 100 mmol), milled potassium carbonate (13.800 g, 100 mmol) and *N*,*N*-dimethylformamide (100 mL) under vigorous stirring at 80 °C for 12 h. Then the mixture was cooled to room temperature, extracted with dichloromethane, washed with water, and dried with Na_2_SO_4_. The crude product was purified by column chromatography with petroleum ether to give light yellow liquid. ^1^H NMR (400 MHz, CDCl_3_) d (ppm): δ 7.89 (d, *J* = 8.4 Hz, 2 H), 7.18 (d, *J* = 8.4 Hz, 2 H), 6.91 (d, *J* = 8.8 Hz, 2 H), 6.83 (d, *J* = 8.4 Hz, 2 H), 5.87 – 5.77 (m, 1 H), 5.05 – 4.95 (m, 2 H), 4.81 – 4.60 (m, 1 H), 4.18 – 4.10 (m, 1 H), 3.91 (t, *J* = 6.4 Hz, 2 H,), 3.85 (s, 3 H), 3.41 – 3.28 (m, 1 H), 2.14 – 2.09 (m, 2 H), 2.04 (s, 2 H), 1.80 – 1.73 (m, 2 H). ^13^C NMR (100 MHz, CDCl_3_) d (ppm): δ 195.51, 163.81, 158.58, 138.54, 130.96, 130.37, 129.51, 128.4, 114.84, 113.88, 79.94, 67.73, 55.54, 41.31, 38.79, 33.43, 28.68, 25.31.

**Synthesis of compound 3:**

Compound **2** (6.04 g, 13 mmol), nitromethane (3.97 g, 65 mmol) and diethylamine (4.76 g, 65 mmol) were dissolved in dry methanol (100 mL) and heated under reflux for 24 h. The solution was cooled down, acidified with 1 M HCl and then extracted with CH_2_Cl_2_/H_2_O. The organic layers were dried over anhydrous Na_2_SO_4_. The solvent was evaporated under reduced pressure. The obtained white solid was used directly in the next step.

**Synthesis of compound 4 and 5:**

Compound **4** and **5** were synthesized according to the previous work.^2^

**Synthesis of compound 6:**

A mixture of compound **3** (3.97 g, 10.0 mmol), compound **5** (5.25 g, 10.0 mmol) and ammonium acetate (38.5 g, 50.0 mol) in butanol (150 mL) was heated under reflux for 48 h. The solution was cooled and the precipitate was filtered. The solid was washed with cold ethanol and isolated as a black solid. The solid was used directly in the next step.

**Synthesis of compound B1:**

Compound **6** (3.00 g, 3.6 mmol) was dissolved in dichloromethane (100 mL). *N*,*N*-Diisopropylethylamine (2.58 g, 20 mmol) was added to the solution, and then the mixture was stirred under ice bath for 2 h. Boron trifluoride etherate (2.84 g, 20 mmol) was added dropwise to the mixture. The solution was quenched with water and then extracted with CH_2_Cl_2_/H_2_O. The combined organic layers were dried over anhydrous Na_2_SO_4_. The solvent was evaporated under reduced pressure. The black solid was obtained. ^1^H NMR (400 MHz, CDCl_3_) d (ppm) δ 8.06 (d, *J =* 8.8 Hz, 2 H), 7.80 (d, *J* = 15.6 Hz, 1 H), 7.61 (d, *J* = 8.8 Hz, 2 H), 7.45 (d, *J* = 15.6 Hz, 1 H), 7.01 – 6.99 (m, 2 H), 6.95 – 6.93 (m, 2 H), 5.91 – 5.81 (m, 1 H), 5.09 – 5.00 (m, 2 H), 4.03 (t, *J* = 6.4 Hz, 2 H), 3.91 (s, 3 H), 2.19 – 2.13 (m, 2 H), 1.88 – 1.81 (m, 2 H), 1.64 – 1.57 (m, 2 H). ^13^C NMR (100 MHz, CDCl_3_) d (ppm): δ 188.81, 163.27, 161.10, 143.93, 138.44, 131.40, 130.71, 130.13, 127.63, 119.41, 114.88, 113.79, 67.95, 55.50, 33.41, 28.61, 25.29.

**Synthesis of P1:**

A mixture of **DMN-acryl** (28.3 mg, 0.09 mmol), MPEG950 (95.0 mg, 0.10 mmol), **B1** (8.8 mg, 0.01 mmol), 2,2-azobisisobutyronitrile (AIBN, 1 mg, 0.006 mmol) and tetrahydrofuran (2.0 mL) was refluxed under nitrogen atmosphere at 80 °C for 24 h. The reaction was then cooled to room temperature. Diethyl ether (100 mL) was poured into the solution to get the emulsion. Then the emulsion was centrifuged (9000 r/min) for 10 min for another 3 times to give **P1** (114 mg, 86%). GPC (THF, polystyrene standard): *M*_n_ = 19674, PDI = 1.20.

**Synthesis of P2:**

A mixture of **DMN-acryl** (31.4 mg, 0.10 mmol), MPEG950 (95.0 mg, 0.10 mmol), 2,2-azobisisobutyronitrile (AIBN, 1 mg, 0.006 mmol) and tetrahydrofuran (2.0 mL) was refluxed under nitrogen atmosphere at 80 °C for 24 h. The reaction was then cooled to room temperature. Diethyl ether (100 mL) was poured into the solution to get the emulsion. Then the emulsion was centrifuged (9000 r/min) for 10 min for another 3 times to give **P2** (106 mg, 84%). GPC (THF, polystyrene standard): *M*_n_ = 21024, PDI = 1.31.

**Synthesis of P3:**

A mixture of MPEG950 (180.5 mg, 0.19 mmol), **B1** (8.8 mg, 0.01 mmol) and 2,2-azobisisobutyronitrile (AIBN, 1 mg, 0.006 mmol) and tetrahydrofuran (2.0 mL) was refluxed under nitrogen atmosphere at 80 °C for 24 h. The reaction was then cooled to room temperature. Diethyl ether (100 mL) was poured into the solution to get the emulsion. Then the emulsion was centrifuged (9000 r/min) for 10 min for another 3 times to give **P3** (172 mg, 91%). GPC (THF, polystyrene standard): *M*_n_ = 16024, PDI = 1.24.

**Synthesis of P1-SO:**

To generate the singlet oxygen loaded polymer **P1-SO**, the dimethyl sulfoxide (DMSO) solution containing **P1** and methylene blue (MB) was irradiated by 660 nm laser (50 mW/cm^2^) at room temperature for 6 h. The solution was bubbled with O_2_ during the whole irradiation. Then MB was removed by dialysis.

**Synthesis of P1 and P1-SO dots:**

2.0 mg **P1** or **P1-SO** was dissolved in 10.0 mL dichloromethane, and then evaporated to form the film. The film was further into 10.0 mL water under the ultra-sonication, followed by the formation of the nanoparticles.

1. **Cell culture and methods**

**Cell culture:**

HeLa cell lines were obtained by the Institute of Biochemistry and Cell Biology, SIBS, CAS (China) and then incubated under 37 °C with 5% CO_2_.

**Cytotoxicity assay in dark:**

The test of cell viability was carried out through the methyl thiazolyltetrazolium (MTT) assays with HeLa cells. Cells were seeded into a 96-well cell culture plate at the density of 10^4^/well at 37 °C with 5% CO_2_ and 100% humidity for 24 h. The cells were incubated with different concentration of **P1** (10 μg/mL, 15 μg/mL, 20 μg/mL, 25 μg/mL and 30 μg/mL), **P1-SO** (10 μg/mL, 15 μg/mL, 20 μg/mL, 25 μg/mL and 30 μg/mL) in dark. Then the cells were treated with MTT (10 μL/well, 5 mg/mL) and incubated for additional 4 h. The medium was replaced by DMSO (150 μL/well). OD570 was monitored by an enzyme-linked immune sorbent assay (ELISA) reader. The following formula was used to calculate the inhibition of cell growth: Cell viability (%) = (mean of Abs. value of the treated group / mean Abs. value of the control group) × 100%.

**Cytotoxicity assay of PDT:**

The test of cell viability of PDT was carried out under 5% and 21% O_2_ with similar experimental condition in dark toxicity test. After adding different concentrations **P1-SO** and **P1** to each well, the cells were irradiated by a 690 nm laser (400 mW/cm^2^) for 6 min.

**ROS detection:**

The HeLa cells were incubated with **P1-SO** or **P1** (20 μg/mL) for 2 h at 37 °C under 5% or 21% O_2_. Then the cells were treated with DCFH-DA (10 μM) at 37 °C for 30 min and then irradiated with 690 nm laser (400 mW/cm^2^) for 6 min. The luminescence of DCF was measured after 20 min by confocal microscopy (λ_ex_ = 488 nm, λ_em_ = 500-540 nm).

**Calcein-AM /PI assays:**

HeLa cells were cultured at 100% humidity 37 °C with 5% CO_2_ for 24 h. **P1** or **P1-SO** (20 μg/mL) was added and incubated for 2 h under 5% or 21% O_2_. The cells were treated with 5 µL PI and 10 µL Calcein-AM in dark for 10 min and then irradiated with a 690 nm laser (400 mW/cm^2^) for 6 min. The images were obtained by the confocal microscopy in green channel for AM (λ_ex_ = 488 nm, λ_em_ = 500-560 nm) and red channel for PI (λ_ex_ = 488 nm, λ_em_ = 600-680 nm) after 1 h.

***In vivo* imaging:**

**P1-SO** or **P1** (300 μg/L, 100 μL) was intravenously injected into the tumor bearing mice. The fluorescence imaging of the mice was obtained by a small animal *in vivo* imaging system (λ_ex_ = 690 nm, λ_em_ = 740 nm) after different times.

***In vivo* singlet oxygen detection of P1-SO:**

The mice were injected with **P1-SO** or **P1** (300 μg/L, 100 μL) through tail intravenous. After 8 h, DCFH-DA was intratumorly injected into the tumor. In the control experiments, the mice were injected with NAC and irradiated with a 690 nm laser (400 mW/cm^2^) for 6 min. The mice were sacrificed after the treatments. The tumor sections were sectioned by the mice and observed by confocal microscopy (λ_ex_ = 488 nm).

***In vivo* photothermal effect of P1-SO** **or** **P1:**

**P1-SO** or **P1** (300 μg/L, 100 μL) was intravenously injected into the tumor bearing mice. The control group of mice was injected with NAC 8 h later, then all the mice were irradiated with a 690 nm laser (400 mW/cm^2^) for 6 min. The temperature change was recorded by a FLIR camera.

**H&E staining:**

Hematoxylin & eosin (H&E) staining of the tumors and organs (heart, liver, spleen, lung, kidney) were obtained for histological examination.

1. **Supplementary figures**

**Figure S1.** The UV-Vis absorption and emission spectra of **B1** (10 μM) in DMSO.

**Figure S2.** Temperature elevation of **B1** at different concentrations under irradiation (690 nm, 400 mW/cm^2^).

**Figure S3.** Absorbance at 243 nm of the mixture of **P2** (200 μg/mL) and MB (10 μM) under different irradiation time (660 nm, 4 mW/cm^2^).

**Figure S4.** Δ_Abs._ of DPBF in the mixture solution of **P2-SO** or **P2** and DBPF under irradiation (690 nm, 400 mW/cm^2^).

**Figure S5.** Δ_Abs._ of DPBF in the mixture solution of **P3** and DBPF under irradiation (690 nm, 400 mW/cm^2^).

**Figure S6.** ROS generation in HeLa cells with DCFH-DA. Cells were incubated with **P1** or **P1-SO** for 2 h under 21 % oxygen under irradiation for 6 min (690 nm, 400 mW/cm^2^).

**Figure S7.** ROS generation in HeLa cells with DCFH-DA. Cells were incubated with **P1** or **P1-SO** for 2 h under 21 % oxygen.

**Figure S8.** ROS generation in HeLa cells with DCFH-DA under 21 % oxygen under irradiation or not (690 nm, 400 mW/cm^2^).

**Figure S9.** MTT assay of **P1** under 21 % oxygen level with and without irradiation (690 nm, 400 mW/cm^2^).

**Figure S10.** MTT assay of **P1-SO** under 21 % oxygen level with and without irradiation (690 nm, 400 mW/cm^2^).

**Figure S11.** Calcein-AM and PI stained HeLa cells were incubated with **P1** and **P1-SO** and then exposed under 690 nm laser irradiation (400 mW/cm^2^) for 6 min under 21% oxygen level.

**Figure S12.** Calcein-AM and PI stained HeLa cells were incubated with **P1** and **P1-SO** and then exposed under 21% oxygen level.

**Figure S13.** Flow cytometry quantification of apoptosis of HeLa cells incubated with **P1** and **P1-SO** under 5% oxygen level without laser irradiation.

**Figure S14.** Photographs of the mice during the whole therapy process.

**Figure S15.** H&E stained of heart, liver, spleen, lung and kidney tissue of different treatment groups.

^1^H NMR spectrum of 2


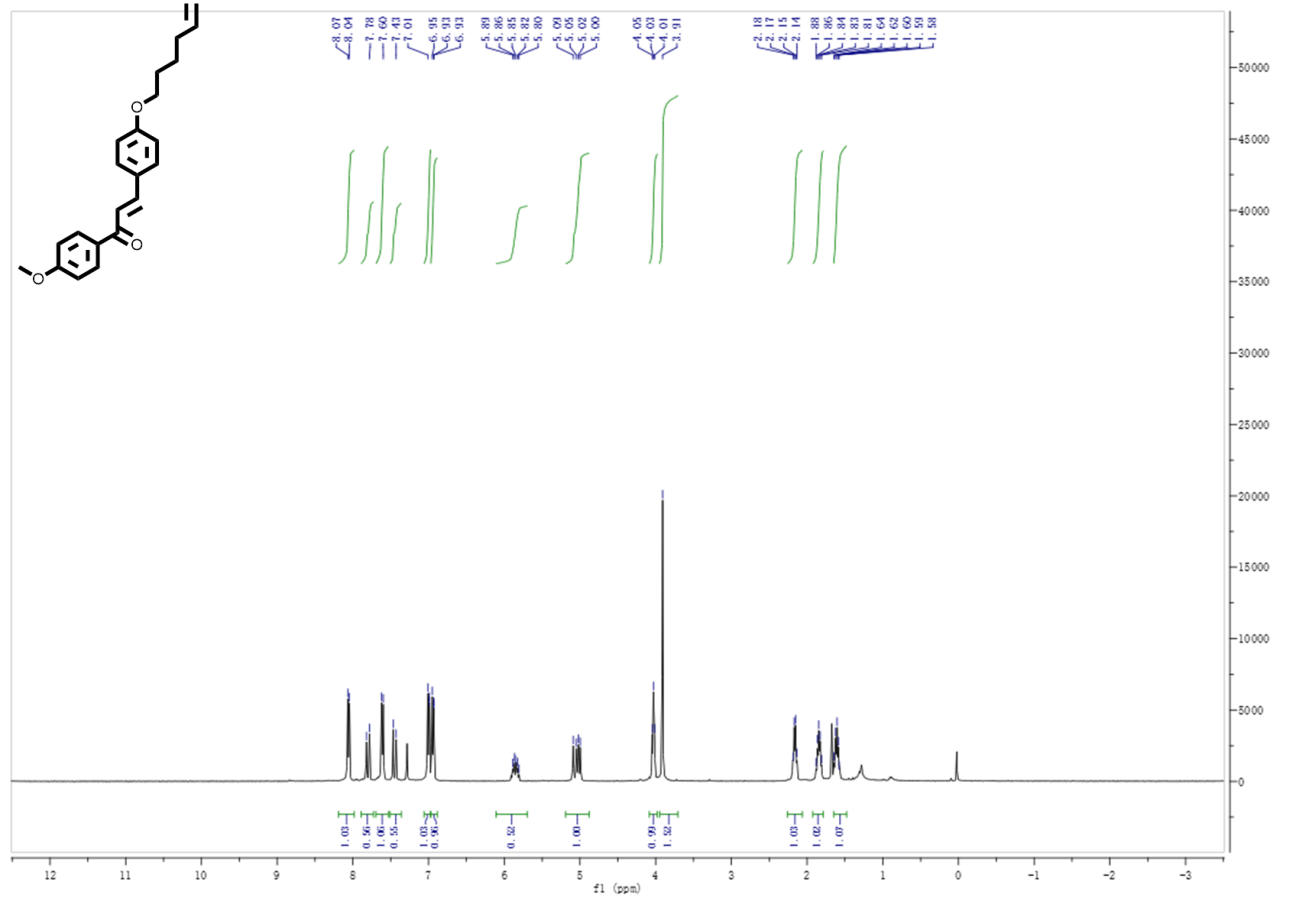


^13^C NMR spectrum of 2


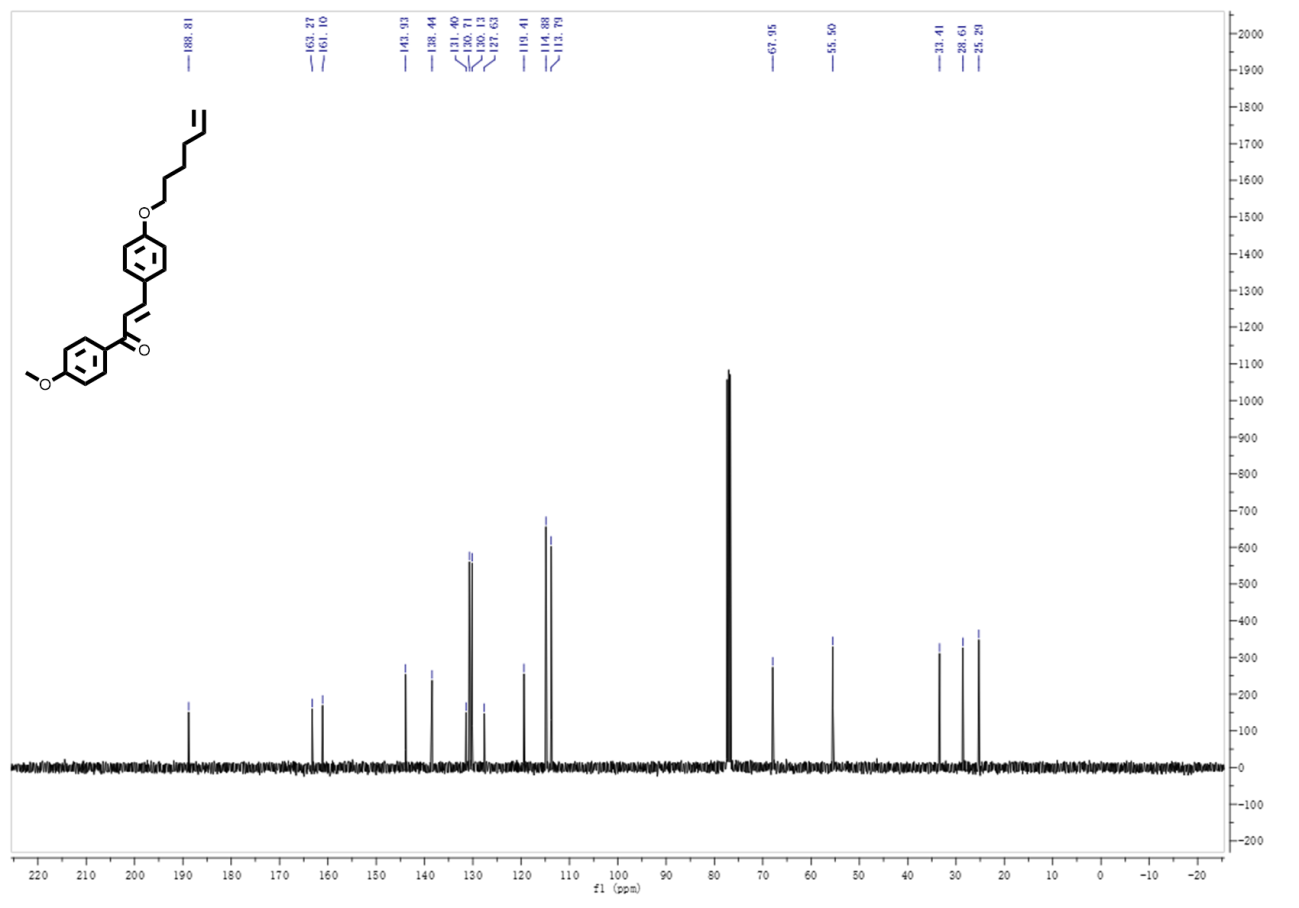


^1^H NMR spectrum of B1

^13^C NMR spectrum of B1

MALIDI-TOF-MS spectrum of B1

^1^H NMR spectrum of P1

^1^H NMR spectrum of P2

^1^H NMR spectrum of P3

**Reference**

(1) Lv, W.; Xia, H.; Zhang, K. Y.; Chen, Z.; Liu, S.; Huang, W.; Zhao, Q. Photothermal-Triggered Release of Singlet Oxygen from An Endoperoxide-containing Polymeric Carrier for Killing Cancer Cells. *Mater. Horiz.* **2017**, *4*, 1185–1189.

(2) Zhao, M.; Xu, Y.; Xie, M.; Zou, L.; Wang, Z.; Liu, S.; Zhao, Q. Halogenated Aza-BODIPY for Imaging-Guided Synergistic Photodynamic and Photothermal Tumor Therapy. *Adv. Healthcare Mater.* **2018**, *18*, 1800606.
